# How the Collaborative Arrangement with CPCLW Facilitated the Efforts of VCS Teams to Support the Wellbeing of Persons Living with Dementia and their Caregivers in their Local Alberta Communities

**DOI:** 10.5334/ijic.9061

**Published:** 2025-05-27

**Authors:** Aleksandra Tymczak, Helen Lightfoot, Blair Wold

**Affiliations:** 1 Alberta Health Services, CA

**Keywords:** VCS teams, governance and collaborative arrangements, wellbeing, communities, multi-sector collaboration, local resources

## Abstract

The work of the 2020–2023 Connecting People & Community for Living Well Health Canada grant initiative focused on determining what contributes to the wellbeing of those living with dementia and their caregivers, across rural Alberta communities, as well as determining what supports the work of the voluntary and community sector (VCS) teams who seek to better support them. These VCS teams included representatives from across local health, social and community sector partners, including local collaboratives. Evaluation findings highlighted the need to support VCS teams to sustain collaborative community-based work and to build and enhance individual and community wellbeing.

## Context and aim

The Connecting People and Community for Living Well (CPCLW) initiative is a Health Canada funded initiative sponsored by Alberta Health Services. At the core of the initiative is how individuals are supported in their community by focusing on individual and collective wellbeing. This shift towards wellbeing moves away from the medical model to one that acknowledges the multiple factors that contribute to peoples’ ability to live well in their own homes and communities. Between January 2020–March 2023, CPCLW supported five rural communities in Alberta to plan, develop and implement community tailored services led by voluntary and community sector (VCS) teams to address the needs of people living with dementia and their caregivers. In the CPCLW work, cross-sector VCS teams are seen as a critical piece of how the community environment shifts to sustain wellbeing. The VCS teams that the CPCLW initiative worked with included partners in social and health care sectors, local organizations (libraries, foundations, etc.) and people with lived experience (persons living with dementia and their caregivers).

In the fall and winter of 2022, five individual focus groups were conducted with VCS teams from the following communities: Drumheller, Innisfail, Stony Plain, Three Hills, and Westlock. The focus group data was then analyzed and reported by the Alberta Health Services Health Systems Knowledge & Evaluation team to understand if and how the collaborative arrangement with CPCLW influenced the VCS teams’ efforts to support the wellbeing of persons living with dementia and their caregivers in their local communities.

## Brief Description of topic at hand

Through a collaborative arrangement, CPCLW facilitated the VCS teams’ efforts to support the wellbeing of persons living with dementia and their caregivers in their local communities in multiple ways. It is important to note that each VCS team was unique, and they experienced support from CPCLW in different ways and to different degrees. CPCLW’s facilitation was observed in three processes of the CPCLW initiative: helping some VCS teams to identify potential cross-sector partners and strengthen their teams; and helping to identify local resources and strengths [1].

## Strengthening the VCS teams

The CPCLW team provided valuable consultation, support, or guidance for some VCS teams as they worked to bring together individuals from across local health, social and community sectors to build and/or organize their collaborative teams to support persons living with dementia and their caregivers. This included helping the communities to identify potential partners and who should be included at the table (especially those with lived experience), along with enhancing some VCS teams’ capacity to build relationships with key sectors at the community level [1]. Across the board, the VCS teams in all five communities were invested in cross-sector collaboration. Their teams not only included core partner organizations in social and health care sectors, but also additional partners involved in community programs and services and those with lived experience [1].

Even though, individuals within the communities had established cross-sector relationships and were continuing to seek additional partnerships prior to the CPCLW initiative, some VCS teams noted that these efforts became more formalized and intentional within the collaborative arrangement provided by the CPCLW initiative. For instance, ongoing meetings with the CPCLW team provided consistent encouragement to continue partnership building efforts, support for helping the VCS team identify partners, and sometimes providing connections with field experts or other community teams who have participated in this type of work [1]. The CPCLW team was also able to fill in a gap on these cross-sector teams by representing provincial level health sector governing organizations such as Alberta Health Services, who are often unaware of VCS teams’ work in the community, despite lobbying and campaigning efforts [1].

## Identifying local resources

It was common that prior to the establishment of the local VCS teams, a systematic approach to coordinating care for people impacted by dementia or for collating information on unmet needs in these communities was lacking. Knowledge and lived experience were often dispersed across various stakeholders in the community [1]. Through the CPCLW initiative helping to strengthen and formalize some of VCS teams, hosting regular meetings, and mapping local resources and community assets, the VCS teams had a better understanding of the full range of skills within partner organizations and gaps in service provision that left some needs unmet. Being part of the CPCLW initiative and having support from the CPCLW team was perceived by several teams to add structure and legitimacy to their efforts, thereby helping to properly utilize their resources [1].

In addition, the strengths-based lens employed by the CPCLW initiative was an important motivator for some VCS teams. It was common for teams to feel overwhelmed by the resource constraints, therefore consistent reassurance from the CPCLW initiative was one way in which teams were encouraged to progress the work forward [1]. The CPCLW team’s structured approach also encouraged the VCS teams to expand their resource searches by identifying new unconventional partnerships and resources that could assist in addressing specific community needs. More generally, the CPCLW approach encouraged the VCS teams to take the time to reflect on their existing strengths and resources [1].

## Discussion and reflection

The evaluation data gathered from the focus groups highlighted several benefits of how the collaborative arrangement with CPCLW facilitated the VCS teams’ efforts to supporting the wellbeing of persons living with dementia and their caregivers in their local communities. These benefits are also echoed in the literature [2,3,4,5,6]. As they worked to help strengthen VCS teams, the CPCLW team was helpful in providing networking support and introductions to partners in social and health care sectors that VCS teams would normally not have been able to easily connect with. The CPCLW team was also active in working to bridge the gap between the VCS teams and provincial level governance organizations such as Alberta Health Services and for advocating of the importance of having individuals with lived experience on the VCS team [2,3,4].

Within the process of identifying local resources, the context-adapted approach and big-picture perspective employed by the CPCLW were useful in prioritizing what specific resources the VCS teams needed during different capacities. Throughout this process, it was also crucial for the CPCLW to provide continual support and encouragement in the face of ongoing challenges and for VCS teams to recognize their strengths [2,4].

Outlining these benefits highlights the instrumental role collaborative arrangements can play in facilitating the work done by VCS teams. Understanding these benefits may increase the interest in existing VCS teams to partner with such initiatives to further their work or enable them to reflect on the supports they do and do not need [4,6].

The feedback gathered via focus groups in combination with the practical experiences of the CPCLW team also illuminated several key lessons that could be summarized as follows: 1) Ongoing and meaningful engagement with lived experience is essential; 2) Trust and relationship building are central to impact wellbeing at both an individual and collective level; 3) Consistent resources are needed; 4) Leveraging existing strengths (e.g., expertise, relationships, and resources) at the provincial and community levels supports sustainability; 5) Building working partnerships across sectors supports sustainability and ensures relevance of approaches [5]. These key lessons are necessary to apply in collaborative arrangements across local, regional and institutional levels to effectively help VCS teams implement care supports in their community [5,6].

## Conclusion

A key to success in addressing local needs and providing care services is cross-sector collaboration of community partners. Bringing together a diverse group of individuals within a VCS team (including partners in social and health care sectors, local organizations and people with lived experience) increases the understanding of the community and local context. Having multiple voices within a VCS team empowers a team to identify what local resources are available, where there are gaps and tailor new or existing programs to ensure wellbeing is improved and sustained.
